# Microbial network for waste activated sludge cascade utilization in an integrated system of microbial electrolysis and anaerobic fermentation

**DOI:** 10.1186/s13068-016-0493-2

**Published:** 2016-04-02

**Authors:** Wenzong Liu, Zhangwei He, Chunxue Yang, Aijuan Zhou, Zechong Guo, Bin Liang, Cristiano Varrone, Ai-Jie Wang

**Affiliations:** Key Laboratory of Environmental Biotechnology, Research Center for Eco-Environmental Sciences, Chinese Academy of Sciences, Beijing, 100085 China; State Key Laboratory of Urban Water Resource and Environment, Harbin Institute of Technology, Harbin, 150090 China; College of Environmental Science and Engineering, Taiyuan University of Technology, Taiyuan, 030024 China; Department of Chemical and Biochemical Engineering, Center for BioProcess Engineering, Technical University of Denmark, Lyngby, Denmark

**Keywords:** Anaerobic digestion, Microbial electrolysis cell, Biogas, Waste activated sludge

## Abstract

**Background:**

Bioelectrochemical systems have been considered a promising novel technology that shows an enhanced energy recovery, as well as generation of value-added products. A number of recent studies suggested that an enhancement of carbon conversion and biogas production can be achieved in an integrated system of microbial electrolysis cell (MEC) and anaerobic digestion (AD) for waste activated sludge (WAS). Microbial communities in integrated system would build a thorough energetic and metabolic interaction network regarding fermentation communities and electrode respiring communities. The characterization of integrated community structure and community shifts is not well understood, however, it starts to attract interest of scientists and engineers.

**Results:**

In the present work, energy recovery and WAS conversion are comprehensively affected by typical pretreated biosolid characteristics. We investigated the interaction of fermentation communities and electrode respiring communities in an integrated system of WAS fermentation and MEC for hydrogen recovery. A high energy recovery was achieved in the MECs feeding WAS fermentation liquid through alkaline pretreatment. Some anaerobes belonging to *Firmicutes* (*Acetoanaerobium*, *Acetobacterium*, and *Fusibacter*) showed synergistic relationship with exoelectrogens in the degradation of complex organic matter or recycling of MEC products (H_2_). High protein and polysaccharide but low fatty acid content led to the dominance of *Proteiniclasticum* and *Parabacteroides*, which showed a delayed contribution to the extracellular electron transport leading to a slow cascade utilization of WAS.

**Conclusions:**

Efficient pretreatment could supply more short-chain fatty acids and higher conductivities in the fermentative liquid, which facilitated mass transfer in anodic biofilm. The overall performance of WAS cascade utilization was substantially related to the microbial community structures, which in turn depended on the initial pretreatment to enhance WAS fermentation. It is worth noting that species in AD and MEC communities are able to build complex networks of interaction, which have not been sufficiently studied so far. It is therefore important to understand how choosing operational parameters can influence reactor performances. The current study highlights the interaction of fermentative bacteria and exoelectrogens in the integrated system.

**Electronic supplementary material:**

The online version of this article (doi:10.1186/s13068-016-0493-2) contains supplementary material, which is available to authorized users.

## Background

The consideration of wastewater and waste sludge as a source of renewable energy is growing, together with the public concerns for future shortage of fossil fuels and the impact on climate change [1]. Anaerobic wastewater treatment processes offer the possibility of a net energy production, rather than aerobic processes that consume energy [2]. Especially, waste activated sludge (WAS), with its abundance of nutrients, has a great potential as an alternative resource to extract value-added products [3–5], even though a long operation time is always required for anaerobic digestion (AD) to achieve an effective carbon removal and energy yield [6].

Recently, a number of studies have suggested that AD can be enhanced by inserting bioelectrochemical systems (BESs) [7, 8] in the anaerobic processes, to enhance acidogenesis [9], hydrogen production [10], or methane production [11–13]. It is well known that microorganisms capable of extracellular electron transfer have a major impact on the natural cycling of carbon and nutrients [14]. Therefore, BESs proved to be highly versatile in terms of potential application for WAS cascade utilization, ranging from energy recovery from organic substrates to product generation and specific environmental niche creation [15]. Characterization of microbial community structure in anaerobic digesters has attracted interest of engineers and researchers, because understanding microbial behavior and interactions is essential to improve the fermentation process [16, 17]. In fact, anaerobic treatment generally requires multiple groups of microorganisms working together to transform primary substrates to energy products such as hydrogen [18] or methane [19]. However, a thorough work in relation to the bacterial community shifts, and especially the impact of fermentation communities to electrode communities, is lacking.

Hydrolysis has always been considered the rate limiting stage in the fermentation of waste solids, therefore most of the studies focused on accelerating sludge hydrolysis using efficient pretreatment strategies, including chemical (such as alkaline [20]) and physical methods (such as ultrasonic [21] or freeze/thaw pretreatment, typically in cold areas [4]). Clearly, the characteristics of different pretreated sludge can also substantially influence the fermentation efficiency and the cascade utilization in the coupled BESs [22–24]. Chemical or physical pretreatments can directly impact the granular size of WAS, leading to the production of various organic compounds [22]. Consequently, digestate from degradation process in digesters presents prime importance for microbes in the recycling of carbon and nutrients [25]. Moreover, the effective diffusion and the biochemical characteristics of carbons and metabolic products can have a significant impact on the electrode biofilm matrix (for example, faster biocatalytic rates were observed under fatty acid-fed conditions) [26]. On the other hand, the efficiency in a BES will also be influenced by the initial anaerobic fermentation community, which will interact with the electrode community, after connecting the BES to a conventional AD system. Therefore, it is very important to evaluate the synergistic effects between fermentative microbes and electrode respiring microbes and the influence of carbon source characteristics. It is also worth noting that metabolic networks and interactions can determine the electrode respiring community development [27].

It is well known that a higher performance of various BESs can be achieved using mixed culture rather than pure culture [28–30]. Though exoelectrogens like *Shewanella* and *Geobacter* are proved to conduct the electron generation and transport in the mixed culture [31], they would not be absolutely dominant compared to other functional members (like *Anaerolinea*, *Bacteroida*, and *Clostridia*) in electrode biofilm. It has been found that interaction between microbes can improve system performance and energy recovery efficiency i.e., when combining *Brevibacillus* with *Pseudomonas* [32], hinting that microbes worked together and contributed to carbon recycling and electron transfer. For example, *Acetoanaerobium* sp. and *Acetobacterium* sp. were reported to be enriched on the bio-electrode of microbial electrolysis cells (MECs) and acetogenesis occurred at a limited degree [33]. *Fusibacter* sp. represented the enriched anaerobic fermentation community, able to utilize carbohydrates and produce acetate and butyrate as end products [34], which represent a favorable substrate to exoelectrogens. In a previous study on anode communities, a complex interaction on carbon degradation was revealed by functional genes (ranging from labile to recalcitrant carbon) [35]. However, synergistic and interactive effects of various communities have been insufficiently examined. In some cases, an unexpected reduction of efficiency and targeted products occured when applying microbial electrolysis to wastewater/sludge treatment [36], though a high energy harvest can easily be achieved from artificial wastewater with pure carbon sources [37, 38].

The relationship between BES and AD (for instance the understanding of their mutual benefits, or system stability enhancement, etc.) has become a debated issue [15], but clearly the effects of newly introduced communities on BES function has to be taken into consideration for further potential application [39]. So far, there were very few reports investigating the characteristics of cascade bioconversion, related to electron recovery, in mixed carbon sources to reveal microbial community interactions between digestate and BES biofilm. Thus, the present study wanted to explore the hypothesis that (i) a proper pretreatment of waste activated sludge can achieve a satisfactory efficiency in terms of final carbon utilization and energy recovery; (ii) sludge cascade utilization can be achieved for hydrogen production in MECs connecting anaerobic fermentation; (iii) fermentative communities formed in AD can have an impact on anode respiring bacteria in batch operation of integrated systems.

## Results and discussion

### Characteristic change of WAS fermentation using different pretreatment

The particle size primarily changed through different pretreatment methods, which substantially influenced the subsequent release and conversion of various organic matter in the WAS. Ultrasonic treatment played the most noticeable effect on sludge structure break and scatter, leading to an average particle size distribution of 29.5 μm (see Additional file 1: Figure S1). Alkaline treatment slightly improved the sludge particle scatter, with an average of 56.3 μm in comparison to 60.8 μm of the control sludge without pretreatment. On the other hand, an obvious increase of particle size up to 387.5 μm was obtained by the freeze/thaw treatment, because flocks were produced after freezing. Consequently, the lysis ratio of increased SCOD to TCOD was 21, 6, and 11 % after alkaline, freeze/thaw, and ultrasonic pretreatment, respectively (see Additional file 1: Figure S2). The freeze/thaw pretreatment was not as effective as other methods on SCOD release, indicating that the flocks of larger particle size were not broken into smaller fragments in a short reaction time [40]. In another report on the effect of sludge pretreatment on sludge characteristics, the disruption of sludge flocks led to the release of intracellular and extracellular materials [41]. Moreover, alkaline (pH 10–12) treatment is known to further enhance the organic release during the pretreatment [42] and favor VFA production in the subsequent fermentation [20, 43]. In our study, an increased amount of soluble organics was released after pretreatment, mainly in the form of carbohydrates, proteins, and volatile fatty acids (VFAs) (see Additional file 1: Tables S1, S2). After 3 d fermentation, SCOD increased from 147 mg/L of the raw sludge to 452 mg/L of the control, 7690 mg/L of the alkaline pretreatment, 1760 mg/L of the freeze/thaw pretreatment, and 3461 mg/L of the ultrasonic pretreatment (see Additional file 1: Table S1). The VFAs were mostly produced by the alkaline pretreatment, which accumulated up to 5300 mg COD/L, accounting for 69 % of total SCOD. The same occurred with proteins, which reached 1749 mg/L, with a 24-fold increase compared to the control sludge without any pretreatment.

In our view, WAS pretreatment initially changed the particle characteristics, which played a major role in the studied process, influencing organic release and fermentative communities during pretreatment, and subsequently affecting the fermentation and VFAs production. Although all sludge pretreatments successfully improved hydrolysis and organic release, the subsequently generated short-chain fatty acids differed in terms of content and concentration, which are known to be important factors affecting the conversion rate and efficiency in bioelectrochemical systems [38]. Clearly, soluble organics play an important role, reducing the accessibility of substrates to bacterial disintegration, or stated differently, the initial particle size can affect the contact surface area, for subsequent bacterial action [44]. Our results showed that ultrasound led to the smallest particle size, followed by a higher acetate production (>80 % of total fermentative products). Alkaline pretreatment could increase the total production of short-chain fatty acids with a high conductivity fermentative liquid. Therefore, it seems to be one of the substantial factors to interact with electrode biofilm communities.

**Fig. 1:**
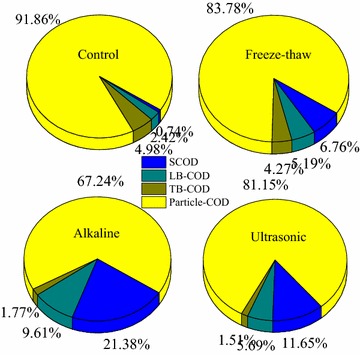
Analysis of main COD distributions in sludge after different pretreatments. The analysis of SCOD, LB-COD, TB-COD and the residual COD in particles after different pretreatments

Furthermore, organics and conductivity of WAS fermentative liquid were the two key factors to the bioelectrochemical communities. To evaluate the influence of pretreatment on COD contribution in different sludge structures, COD was divided into four parts: soluble SCOD, loosely bound extracellular polymeric substances (LB-EPS), tightly bound extracellular polymeric substances (TB-EPS), and residual particles (Fig. 1). The alkaline pretreatment effectively released ~25 % particle organics (compared to the control) into SCOD (~21 %). A small part of particles (4 %) with reduced TB-EPS (~3 %) were converted into LB COD (~7 %). However, COD contribution was reduced to less than half of SCOD (6–11 %) from particles, when using the freeze/thaw or ultrasonic pretreatment. The SCOD of VFAs reached the peak accumulation during fermentation before methane production started, under the conditions of this study [45, 46]. The release of soluble matter also increased conductivity of fermentation solution. Even during fermentation without any pretreatment, there was a slight increase from 1.2 to 1.4 mS/cm in sludge fermentation liquid (SFL) (see Additional file 1: Table S1). Conductivity was further increased to 1.96–2.63 mS/cm by the freeze/thaw and ultrasonic treatment respectively, which matched the increasing trend of SCOD and inorganic ion release. The alkaline addition, using NaOH, highly enhanced the conductivity, reaching up to 6.23 mS/cm, which was almost close to 50 mM PBS (Phosphate buffer solution, pH 7.0) used for MEC reactor setup [35]. A high conductivity is to be considered potentially beneficial to electron transport in the following bioelectrochemical process [47]. Besides the additional alkaline contribution, organics and ion release from WAS improves during the pretreatment and is further enhanced during the fermentation. A previous study showed that the limiting factors, at the anodic biofilm, change from potential limitations at low conductivity, to dual potential and carbon source transfer limitations at a moderate conductivity, and to only mass transfer limitations at high conductivity [48]. A low conductivity (<1 mS/cm) was observed in common AD effluent after organic removal and biological treatment, moreover, a higher external voltage was required when connecting BES after AD to achieve biofuels [49]. In this respect, pretreatment is an important and flexible tool to regulate the performance of BES and AD integrated process, which would determine the total efficiency on waste treatment and biofuel recovery.

### Pretreated SFL utilization and hydrogen production in MECs

The setup performance of the 15 MEC replicate reactors, before fueling SFL (see Additional file 1: Figure S3). The average coulombic efficiency was steadily around 92.2 ± 6.5 % in all replicates, with an average peak current of 3.75 ± 0.22 mA. The COD removal efficiency of acetate reached 86.8 ± 2.1 %. The 15 MEC reactors showed similar conversion efficiencies to hydrogen, with 3.3 ± 0.5 mol H_2_/mol acetate and a hydrogen production rate of 1.36 ± 0.26 mL/mg COD (1.46 ± 0.28 mL H_2_/mL reactor/d). Twelve reactors were randomly divided into four groups (three replicates each), to be fed with SFL obtained from the different pretreatment methods.

**Fig. 2:**
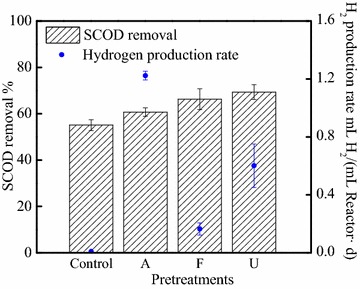
SCOD removal and hydrogen production rate in MECs fed with different sludge fermentative liquids. *Control* sludge without treatment, *A* Alkaline,* F* freeze/thaw,* U* ultrasonic pretreatment

The pretreated sludge properties determined the subsequent fermentation process, leading to various levels of acidification, organic contents, and production rates (see Additional file 1: Figures S4, S5). The highest amount of VFAs was produced during the 3rd day fermentation of alkaline pretreated WAS, containing 2225.81 mgCOD/L and accounting for 42 % in total VFAs (see Additional file 1: Figure S4). There was 1077.25 mgCOD/L acetate produced in ultrasonic pretreated WAS, while still accounting for 41 % of total VFAs. The lowest amount of VFAs was observed with the freeze/thaw-pretreated WAS, though still showing a 3.8-fold increase compared to WAS without pretreatment. In all pretreated SFL, more VFAs were firstly utilized in MECs, showing a similar removal of around 70 % (see Additional file 1: Figure S5). Over 95 % of acetate and butyrate were utilized in alkaline SFL and ultrasonic SFL, while only ~85 % acetate and butyrate were removed in freeze/thaw SFL. Differently, a much higher percentage of propionate (removal amount was really low) was removed in freeze/thaw SFL than others at the same time. As a result, hydrogen production rate differed in MEC reactors, based on acid types and concentrations that were produced [38]. Previous results showed that pretreatment methods are very important to release organics and enhance degradation of various carbon sources from WAS [46, 50]. Probably, the cascade utilization of SFL could be regulated according to composition in VFAs, proteins, and polysaccharide [22, 51], while the energy recovery changed when the suitable organic compounds were degraded. It is therefore likely that the alkaline treatment performed best energy gains (Fig. 2) thanks to the high conductivity (increased from 2.1 ± 0.2 mS/cm for raw sludge to 3.5 ± 0.3 mS/cm for alkaline pretreatment) [51, 52] and SCOD, as well as high COD removal.

The current generation varied among different pretreated and fermented sludge (see Additional file 1: Figure S6). The highest peak current reached ~3.7 mA, when feeding with the alkaline pretreated SFL, which showed the highest acetate production of 2200 mg COD/L, as well as an enhanced conductivity. The peak current dropped to 2.5 mA for ultrasonic and 1.8 mA for freeze/thaw condition. The lowest current was only 1.0 mA, using SFL produced from raw WAS without pretreatment. The SCOD removal was slightly different in different SFL, with 61 ± 2 % for alkaline, 66 ± 5 % for freeze/thaw, and 69 ± 3 % for ultrasonic pretreatment in MECs (Fig. 2). However, the hydrogen production rate was quite different. The alkaline pretreated SFL achieved the highest hydrogen production of 1.22 ± 0.03 mL H_2_/mL reactor/d (compared to 1.46 ± 0.28 mL H_2_/mL reactor/d before fueling with SFL). The ultrasonic pretreated SFL was converted into hydrogen with a rate of 0.60 ± 0.15 mL H_2_/mL reactor/d. The freeze/thaw pretreatment, instead, was not able to effectively improve the cascade utilization of WAS, compared to raw sludge.

Clearly, organic products were the result of metabolic activities of the microbial community, which was characterized by different composition and structure during fermentation. Previous studies showed that with the proper enrichment of microbial communities, anaerobic processes can be improved and perform more efficiently [53]. In this study, the solid granular sludge changed based on different pretreatment methods. Although SCOD was increased and consequently converted to more hydrogen in MECs, the hydrogen yield was reduced by ~16 % when influent was changed from artificial wastewater (acetate, ~1140 mg COD/L) to SFL (acetate, ~2225 mg COD/L accounting for 29 % of SCOD in the alkaline pretreatment) with fermentative communities. Moreover, hydrogen yield was reduced by ~59 % when feeding the ultrasonic pretreatment SFL (acetate, ~1080 mg COD/L accounting for 31 % of SCOD). It has been pointed out that further increases in organic loading do not vary hydrogen production significantly [54, 55]. Therefore, it is likely that MEC performances changed in relation to anodic community structure, which interacted with dominant fermentative communities and organic compounds produced [56].

### Methane production and archaea community change in integrated system

**Fig. 3:**
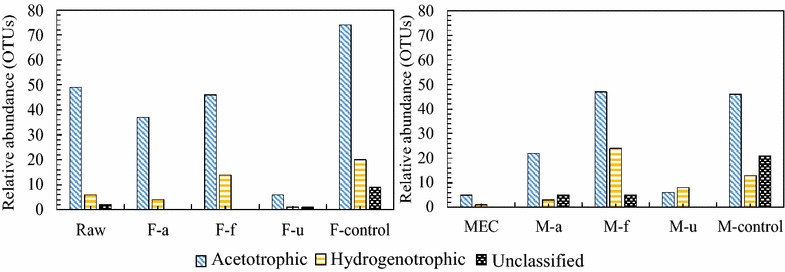
Relative abundance of Archaea communities based on OTUs.* F samples* fermentative liquid after treatment,* M samples* biofilm in MEC reactors (detailed sample legends see Fig. 2)

Methane production was detected in all MEC reactors after 2 weeks feeding SFL, however, the methane production rate was fluctuating, not being comparable among different pretreatments over all batch operations (data not shown). Although methane production was not substantially increased over 1 month (as we evaluated previously [57]), the MECs feeding SFL without pretreatment presented the highest methane production over all other conditions, together with the highest amount of acetotrophic methanogens (*Methanosaeta*), both in control SFL and MEC biofilms fed with control SFL (Fig. 3). It was interesting that the lowest amount of archaea were detected under ultrasonic pretreatment, leading to the lowest growth in MECs as a result. Compared to methanogens in initial biofilms fed with acetate (MEC sample), it seem that acetotrophic methanogens were substantially enriched to anode biofilm in all conditions. But hydrogenotrophic methanogens were further enriched with higher amounts than in SFL feeding, as shown in freeze/thaw and ultrasonic pretreatment, including *Methanocorpusculum*, *Methanosphaerula*, *Methanoregula*, *Methanospirillum*, *Methanobacterium*, and *Methanobrevibacter*. The extra hydrogen generation from MECs can favor hydrogenotrophic methanogens in anaerobic condition [57]. The H_2_ produced in a single chamber MEC can be lost through methanogenesis, which causes energy loss in the system [22, 51, 52].

### Microbial community structure and anodic biofilm community shift in integrated process

A total of 244,761 raw sequences were analyzed over all community samples (Additional file 1: Table S2). Operational taxonomic units (OTUs) at 3 % distance were the most detected ones in raw WAS (5002), with the highest diversity (Shannon index 6.96), while being the least detected in the startup anode biofilm using acetate (2341), showing a reduced diversity (Shannon index 5.39) (see Additional file 1: Figure S7). Similar results were observed from ACE (abundance-based coverage estimator) and Chao1 indices (see Additional file 1: Table S3). Interestingly, microbial community diversity in the SFL decreased, indicating that specific fermentation bacteria were enriched and became dominant. On the other hand, an increase of diversity was detected in anodic biofilm communities, after initial MECs were connected to sludge fermentation (with or without pretreatment) for several days, showing an interactive effect of fermentative communities on initial anodic communities, thus leading to subsequent changes in MEC reactor performances.

After fermentation, the unpretreated sludge showed similar community structure to raw sludge (see Additional file 1: Figure S8). The most abundant phylum in SFL was *Proteobacteria*, accounting for 36.7 % in the control, 40.0 % in the alkaline pretreatment, 28.7 % in the freeze/thaw pretreatment, and 54.8 % in the ultrasonic pretreatment, over all microbial communities. Seemingly, sludge fermentation after ultrasonic pretreatment mostly increased *Gammaproteobacteria*. In comparison to the control sludge, *Firmicutes* (*Bacilli* sp. and *Clostridia* sp.) were all increased in SFL of the pretreated sludge. *Bacteroidetes* was the third most abundant community in the SFL.

**Fig. 4:**
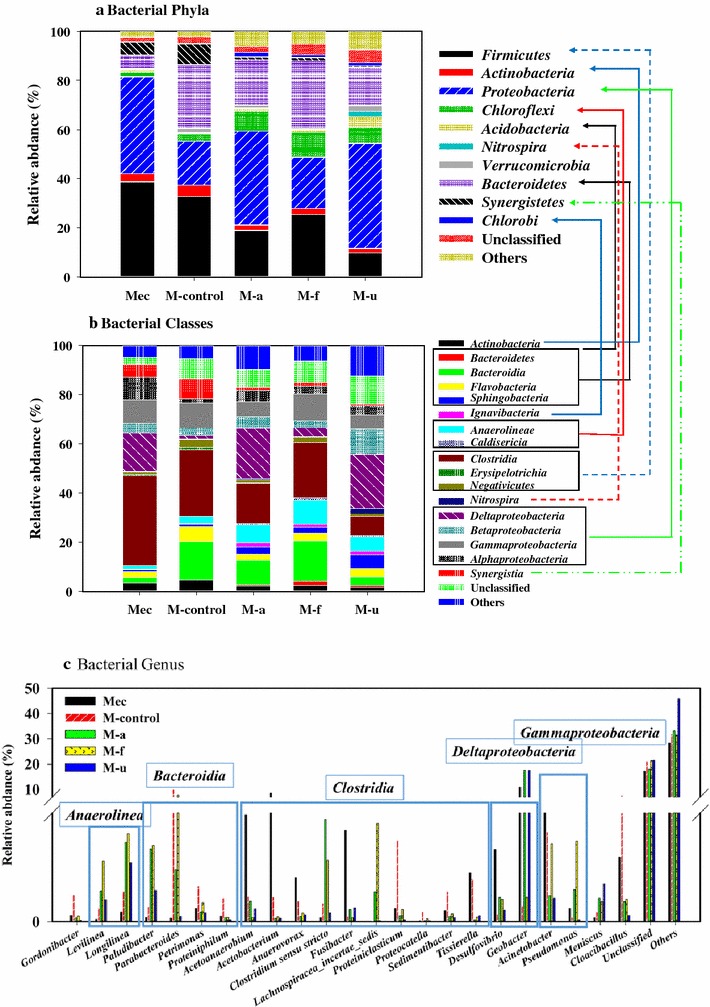
Taxonomic classification of 16S rRNA gene sequences of bacterial communities of anode biofilm at the phylum (**a**), class (**b**), and genus (**c**) levels. Relative abundance was defined as the number of sequences per sample.* Mec* MEC initially fed with acetate,* M-control* SFL-fed MEC without treatment (control treatment),* M-a* MEC fed with alkaline treated SFL,* M-f* MEC fed with freeze/thaw treated SFL,* M-u* MEC fed with ultrasonic treated SFL

When MECs were connected to the fermentation process, anodic biofilm composition obviously changed, compared to the original communities established using acetate (Fig. 4). *Desulfovibrio* [58] and *Geobacter* [14] (*Deltaproteobacteria*), responsible for electron transfer between bacteria and electrode, represented the key functional community. *Geobacter* was the most detected genus of the anode biofilm, in the case of reactors fed with acetate (startup MECs), and further increased after feeding with alkaline pretreated SFL, as well as the ultrasonic pretreated SFL (with a corresponding high energy conversion achieved in these reactors). The MEC fed with the freeze/thaw-pretreated SFL, instead, showed low abundances of *Desulfovibrio* and *Geobacter,* which was similar to the control SFL. On the other hand, compared to other treatments, freezing-thaw SFL led to an increased abundance of *Pseudomonas* in the anodic community. Moreover, large particles of organics in SFL led to enrichment of fermentative communities in the anode biofilm, including *Anaerolinea* (*Levilinea* and *Longlinea*), *Bacteroida* (*Paludibacter* and *Parabacteroides*), and *Clostridia* (*Proteinilclasticum*, *Proteocatella*, and *Sedimentibacter*). Compared to the original anode biofilm, four genera of the class *Clostridia* (namely *Acetoanaerobium*, *Acetobacterium*, *Anaerovorax*, and *Fusibacter*) decreased in all SFL-fed MECs.

**Fig. 5:**
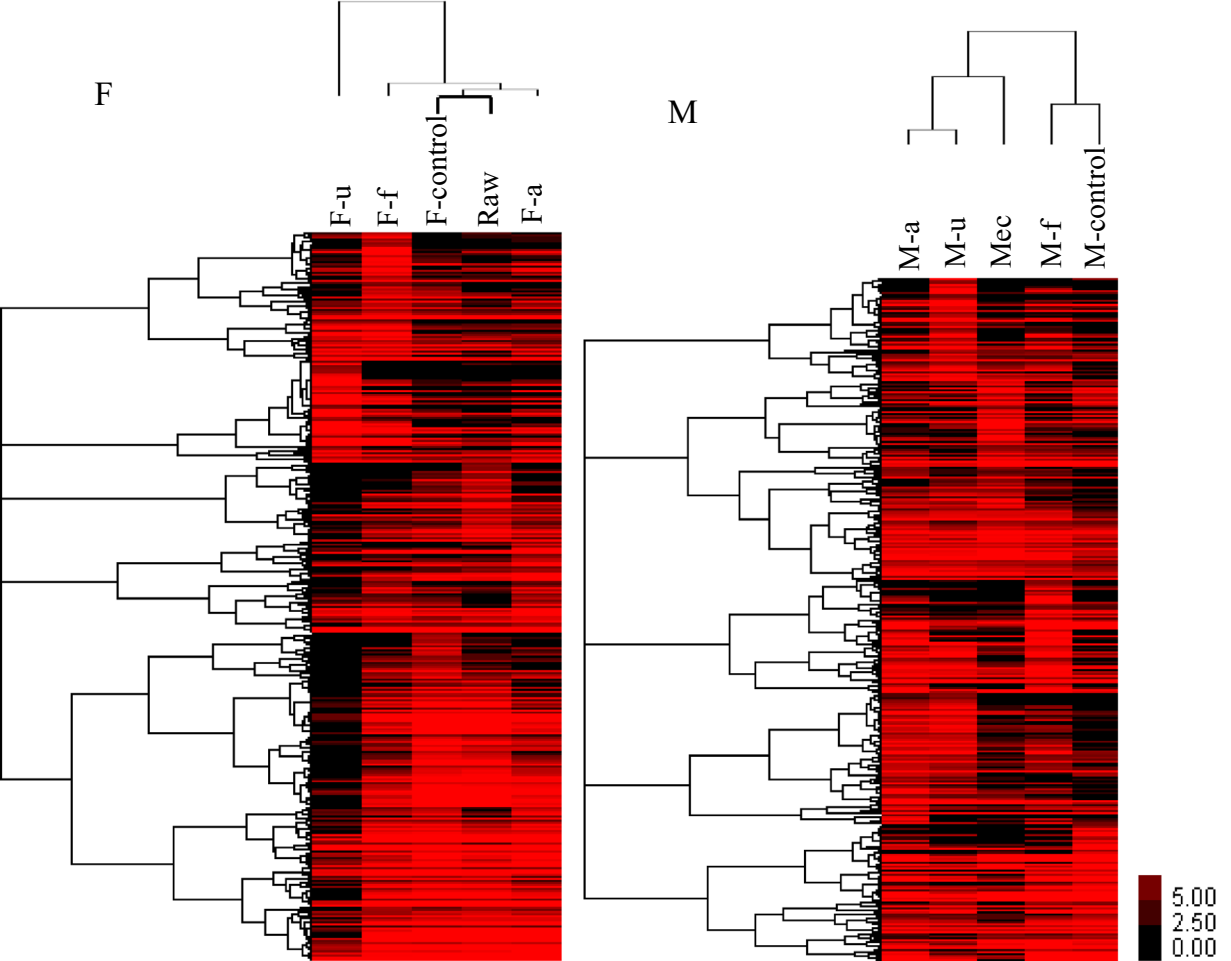
Hierarchical cluster analysis of bacterial communities from sludge fermentative liquid (*F*) and MEC biofilm (*M*). The OTUs of* y-axis* were ordered by phylum (3 % distance). Sample communities were clustered based on complete linkage method. The* color* intensity of scale indicates relative abundance of each OTU read (sample legends see Fig. 2)

Moreover, hierarchical cluster analysis clearly showed that SFL communities varied, depending on the different treatment method (Fig. 5, F-samples). The bacterial community structure of the control SFL (F-control) without pretreatment changed less, compared to the raw sludge (Raw), while the ultrasonic pretreatment led to the greatest difference in community structure in the SFL (F-f). Figure 5 (M-samples) showed how various SFL communities had different impacts on the change of the anodic biofilm communities, after combining fermentation and microbial electrolysis process. Interestingly, anodic biofilm communities were similarly grouped among the original MEC (Mec), MECs fed with the alkaline pretreated SFL (M-a), as well as MECs fed with ultrasonic pretreated SFL (M-u). Regarding the gas production, they performed much higher hydrogen yield than the SFL-fed control or the freeze/thaw pretreatment, which had a low hydrogen conversion, below 0.2 mL/mg COD, and peak current below 1.0 mA.

**Fig. 6:**
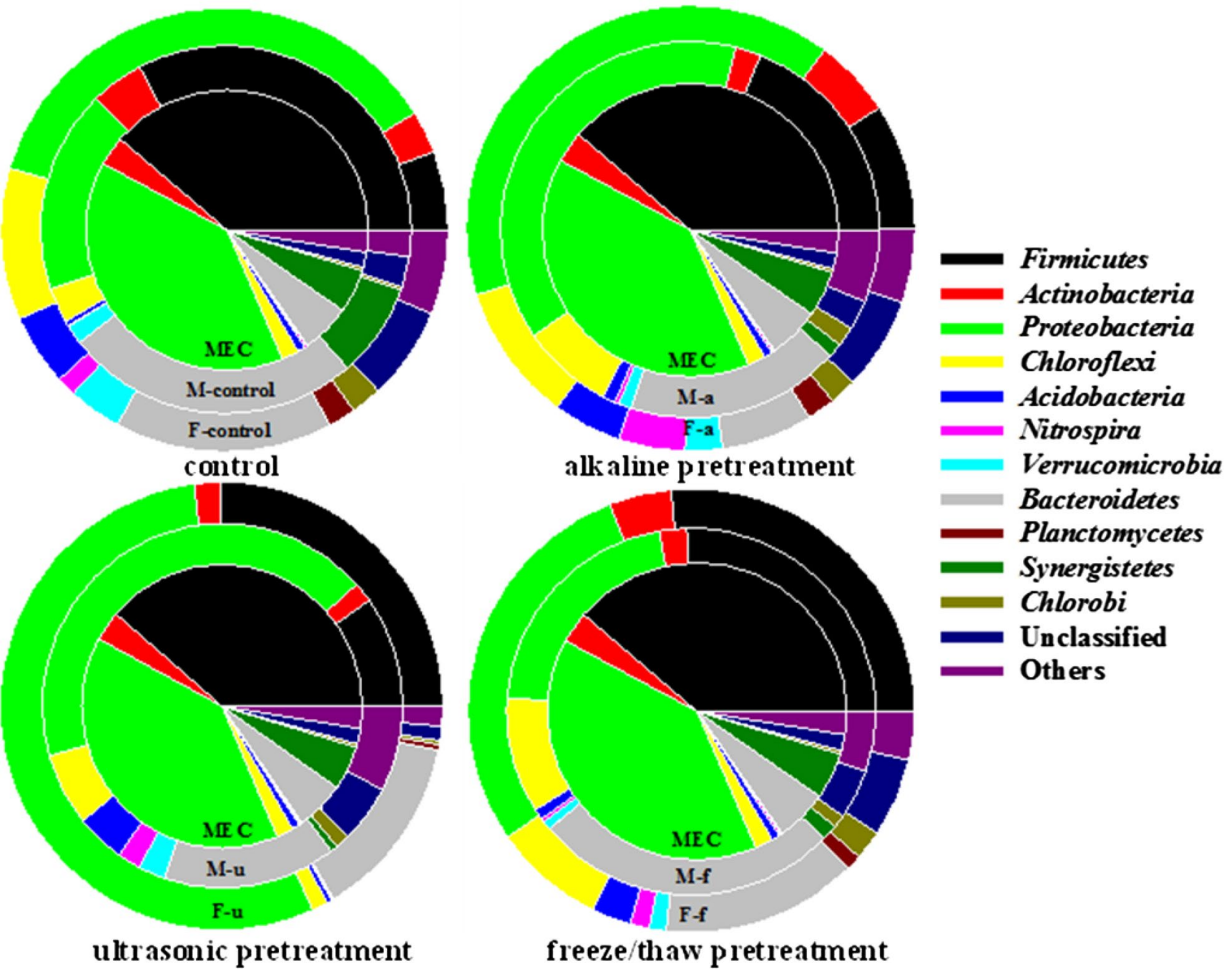
Community change and linkage during sludge cascade utilization (from fermentation to MEC) after different sludge treatments. Bacterial communities based on OUT (3 % distance), and the taxonomic identification of OTUs at phylum level, during sludge cascade utilization of alkaline treated SFL; anode biofilm communities (MEC) changed into a new biofilm community (M-a) after feeding SFL (communities of F-control)

Bioelectrochemical communities were highly enriched in dominant functional groups related to *Proteobacteria* (63 %) and *Firmicutes* (25 %) when feeding acetate during reactor setup, inoculated with activated sludge (Fig. 6). They played the primary function of electron transfer and substrate degradation, with great potential on complex carbon utilization, as already suggested from a functional genes’ perspective [35]. The most abundant genera for extracellular electron transfer were *Geobacter*, *Desulfovibrio*, and *Acinetobacter*, belonging to *Proteobacteria*. *Geobacter* species are considered as the most efficient exoelectrogens in bioelectrochemical systems [59]. *Desulfovibrio* and *Acinetobacter* species are dissimilatory metal-reducing bacteria involved in contaminant degradation and metal reduction, outside the cell membrane [60, 61]. In *Firmicutes*, three genera of *Clostridia* were detected, namely *Acetoanaerobium* (5.9 %), *Acetobacterium* (8.5 %), and *Fusibacter* (5.0 %). They are supposed to play an important role in carbon recycling for anode respiring bacteria, as previous studies have shown that some *Firmicutes* may closely live with anode respiring bacteria, when fed with fermentative substrates [62].

### Microbial community network on exoelectrogenic and fermentative communities

**Fig. 7:**
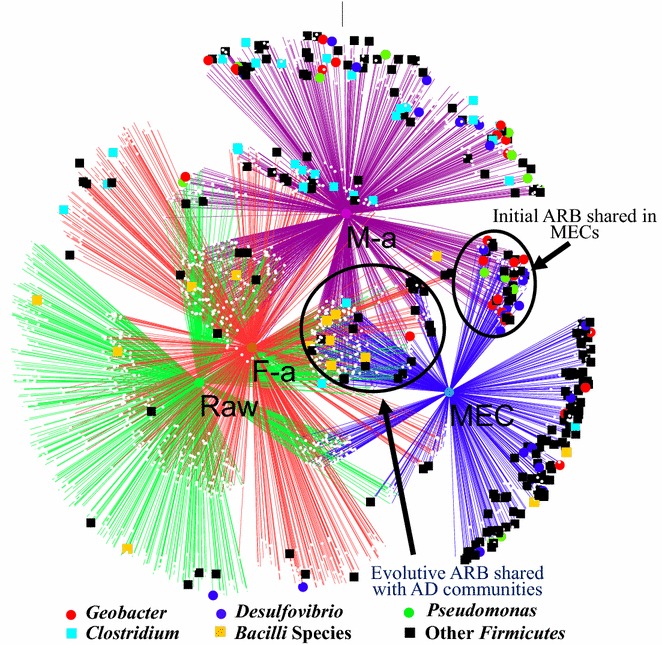
Network of communities based on OTUs in sludge cascade utilization, through alkaline pretreatment. Electron transfer in integrated system was conducted by part of the initial anode respiring bacteria (ARB) and part of the evolutive ARB, interacting with fermentative communities.* M-a* MEC fed with alkaline treated SFL,* F-a* Fermentative solution of alkaline pretreatment

Based on the discussion above, a network representing the community change and linkage was constructed (Fig. 7), taking alkaline treatment samples as example. MEC biofilm was inoculated from raw sludge (Raw), then raw sludge was pretreated to produce fermentation liquid as feedstock for the MECs. During the cascade process, MEC biofilm interacted with fermentative communities to form a new MEC biofilm. The SFL primarily led to an increase in abundance of *Bacteroidetes* and *Chloroflexi* in anodic communities, thus reducing the abundance of *Proteobacteria*. Even though the phylum *Bacteroidetes* was commonly detected in bioelectrochemical system communities, few studies pointed out their negative impacts on electron transfer efficiency [33]. On the other hand, *Bacteroidetes* can be further enriched (over *Proteobacteria*) in an open-circuit BES to convert substrates, thus competing with anode respiring bacteria for power output [63]. Lately, it was highlighted that *Bacteroidetes* can be easily enriched in BES when supplemented with other electron acceptors (NO_3_^−^) [64], thus potentially enhancing an electron flow that is separated from the energy yield in MECs. *Chloroflexi* also represented an enriched phylum in the open circuit, and it is usually predominant in anaerobic digester sludge. *Bacteroidetes* increased to 25.9 %, compared to 5.6 % in the initial setup MECs, with high coulombic efficiency and H_2_ yield. *Proteobacteria,* on the contrary, decreased to 17.8 % in SFL control MECs, although they increased to 26.8 % by feeding with freeze/thaw-pretreated SFL. More specifically, *Gammaproteobacteria* and *Deltaproteobacteria* were partially decreased, while *Betaproteobacteria* partially increased, thus leading, as a result, to a lower efficiency of electron transfer and hydrogen recovery.

MECs fed with alkaline pretreated SFL showed the best energy yield and shared the largest community of *Geobacter*, *Desulfovibrio*, *Pseudomonas*, and *Clostridium,* with initial MECs (Fig. 7). Simultaneously, *Firmicutes* substantially decreased, including *Acetoanaerobium* (1.1 %), *Acetobacterium* (0.2 %), and *Fusibacter* (0.3 %). Some of the anaerobes (>20 %), i.e., the class *Bacilli* (*Pasteuria* and *Lactococcus*) and *Clostridia* (*Fusibacter*, *Anaerovorax*, and *Proteiniclasticum*), were clearly enriched on electrode biofilm by SFL feeding, suggesting a synergistic effect with exoelectrogens to degrade complex organic matter [35] (like *Lactococcus* producing soluble electron shuttles to promote electron transfer between cells and the electrode surface [65]). Some genera belonging to *Clostridia* (*Acetobacterium* and *Acetoanaerobium*) (<10 %) were probably enriched in MECs by the availability of products such as hydrogen (electron). However, their function on carbon/electron recycling seems to be very limited in various BES systems [33], which would suggest only a limited hydrogen loss (or over 100 % coulombic efficiency) in MECs fed with acetate [66], carbohydrate [67], or fermentation liquid [51]. It is worth noting that a high protein or polysaccharide but low fatty acid content would lead to the dominance of *Proteiniclasticum* and *Parabacteroides* (increased by >10 %), which are able to produce VFAs as end products from fermentation [68, 69]. Thus, part of the microbial communities did not function on extracellular electron transfer; however, they were maintained in the fermentation niche of electrode biofilm, where they could provide labile products for electrode respiring bacteria. A substantial reduction in current and hydrogen recovery (Alkaline vs. Ultrasonic) was observed when introducing SFL with an increased abundance in *Proteiniclasticum* (Alkaline vs. Ultrasonic genus level in Additional file 1: Figure S8). *Proteiniclasticum* reached 11.4 % in the ultrasonic pretreated SFL, but only 0.4 % in the alkaline pretreated SFL. In fact, MECs presented a similar COD removal (61 and 69 %) but coulombs were reduced by 31 % and hydrogen production by 50 %.

Alkaline pretreatment could provide more short-chain fatty acids and higher conductivities than other pretreatments. These two aspects are known to favor mass transfer in anodic biofilm [48]. *Geobacter* increased in both, alkaline and ultrasonic pretreated SFL, in which accumulated VFAs were higher than 2500 mgCOD/L (with acetate reaching >1000 mgCOD/L). It appeared that some species, belonging to *Parabacteroides*, *Clostridium*, and *Pseudomonas*, were potentially enriched more in alkaline SFL with higher SCOD and conductivity, compared to ultrasonic SFL. But a delayed fermentation process on raw WAS, as well as freeze/thaw-pretreated WAS, substantially led to different fermentative communities in the anode biofilm (such as *Parabacteroides* and *Proteinilclasticum)*, which produced little VFAs for anode respiring bacteria. In this situation, long time would be required to WAS cascade utilization, with a slow fermentation and inefficient electron generation in BES.

## Conclusions

WAS, rich in organic carbon, can be used as an alternative bioresource, and energy recovery can be potentially improved when combining proper pretreatment method and novel AD technology. During the cascade utilization of WAS, organic removal could be improved by various pretreatment methods, while energy recovery of MEC was impacted by fermentation liquid and microbial community composition. A smaller particle size of WAS after pretreatment substantially favored the fermentation process and formation of suitable byproducts for BES. Alkaline pretreatment gave the highest VFAs production, achieving the most effective energy recovery in the MECs with abundance of *Geobacter* >10 %. Energy yield of electron transfer in integrated system was influenced, on one side, by the initially formed exoelectrogen community, but also by the newly formed one, after interacting with fermentative communities. A high protein or polysaccharide (but low fatty acids) content led to the dominance of *Proteiniclasticum* and *Parabacteroides*, which had a delayed contribution to extracellular electron transfer.

## Methods

### Pretreatment of WAS and short fermentation process

The WAS was collected from the secondary sedimentation tank of Wenchang municipal WWTP in Harbin, China. The sludge was concentrated by settling for 24 h and moving the water layer away, then stored in the refrigerator at 4 °C. The large particles were separated through the 40 mesh sieve. In order to facilitate comparison among different treatments, the volatile suspended solid (VSS) content of the WAS used in this study was adjusted to 14 g/L. Three kinds of pretreatment methods were used, prior to fermentation: (a) alkaline treatment, in which to the pH was adjusted to 10 (by NaOH) and the sludge reacted for 20 min at 81 °C [45]; (b) freeze/thaw treatment, by freezing the sludge at −20 °C for 72 h and then thawing it at room temperature, before use [70]; (c) ultrasonic treatment, in which the sludge was treated for 10 min with 0.5 kW/L energy density, using 40 kHz bi-frequency ultrasound; (d) the control, without any treatment of the raw sludge [46].

After pretreatment, the particle size distribution was analyzed with a particle analyzer (Mastersizer 2000 Malvern) with a Hydro 2000MU dispersing unit and detected by means of laser diffractometry (within approximately 30 s). The following parameters were set: refractive index (RI) values for particles and basis solution (water, 20 °C) were 1.52 and 1.33, respectively and the measurement cycle is 10 s. The analysis was repeated three times, and the average readings were obtained by Mastersizer 2000 software. The output of the measurements is depicted in a graph of volume (%) against particle size (μm) within a range from 0.02 to 2000 μm. The stirrer and pump speed were kept at 600 rpm, which is the minimum pump/stirrer speed of the instrument, to eliminate the potential damage to the sludge flocks [71]. The WAS fermentation took place in sealed glass bottles (effective volume of 500 mL, Sichuan Shubo CO., China). Each bottle contained 350 mL WAS. All bottles were flushed with nitrogen gas for 10 min to remove oxygen. The bottles were stirred in an air-bath shaker at 35 ± 2 °C for 3 days to achieve a good accumulation of VFAs [45]. The supernatant was taken from SFL after settling overnight (12 h), and used as carbon source for MECs.

### MECs reactor setup and operational conditions

Fifteen single chamber MEC reactors were set up (see Additional file 1: Figure S9). The reactors were made of polycarbonate. The effective volume was 40 mL, including a 28 mL chamber (3 cm diameter × 4 cm length) and a 10 mL injection syringe as a gas collection tube (valid volume 12 mL). The anode was a graphite brush (1.6 cm diameter × 8 cm; 0.22 m^2^ surface area). The cathode was made of carbon cloth (YW-50, YiBang Technology Co., Ltd., China), covered with a Pt catalyst layer (0.5 mg Pt/cm^2^ in one side) [51].

The reactors were firstly inoculated with the sludge from the Wenchang municipal WWTP and were fed with acetate (1500 mg/L) as carbon source, in 50 mM phosphate buffer solution (PBS, containing NH_4_Cl 0.31 g/L, KCl 0.13 g/L, NaH_2_PO_4_·2H_2_O 5.618 g/L, Na_2_HPO_4_·12H_2_O 6.155 g/L, pH 7.0 ± 0.5, 7.3 ± 0.3 mS/cm) [35, 51]. The external voltage was 0.80 ± 0.01 V. All the reactors kept running stably, in 24 h batch operation, for at least 10 cycles till all replicates performed similar current, COD removal, and gas production. Twelve reactors were picked out of the 15, and then every three reactors were set as replicates, which were randomly divided into four groups for the test of different sludge fermentative liquid, obtained from different treatment, as described above. The SFL for MECs was discharged and refilled at the end of every 3 d batch cycle over 1 month. A flow schematic representation of experimental methodology and reactor setup is shown in (see Additional file 1: Figure S10).

### Analysis and calculation methods

The currents were automatically monitored (Acquisition system; Keithley Instrument, US) through a 10 Ω resister. The gas was collected in a gas bag (50 mL; Cali5-Bond; Calibrated Instrument Inc, US). Gas composition was analyzed by a gas chromatograph (Fuli, GC9790; Zhenjiang Instrument Inc, China), with a packed column [51] (TDX-01; 2 m length) and equipped with a TCD detector. The volume of gas was measured by a glass syringe.

LB-EPSs and TB-EPSs were extracted according to previous studies [72, 73], and were modified appropriately. The specific method was: firstly, 10 mL sample was centrifuged at 4000*g* for 10 min, the supernatant was filtered with 0.45 μm cellulose nitrate membrane filters, and the filtrate was serviced as dissolved organic matters (DOMs). Secondly, the residue in centrifuge tube was treated according to the EPS extraction method for LB-EPSs [72]. The filtrate was processed as the LB-EPSs. Finally, after LB-EPSs extraction, the residue in centrifuge tube was treated according to the EPSs extraction method for TB-EPSs and filtrate was regarded as the TB-EPSs.

The short-chain fatty acids (SCFAs) were recorded as the sum of acetic (HAc), propionic (HPr), *n*-butyric (*n*-HBu), *iso*-butyric (*iso*-HBu), *n*-valeric (*n*-HVa), and *iso*-valeric acids (*iso*-HVa). SCFAs were analyzed by a gas chromatograph (Agilent 4890; J&W Scientific, USA) equipped with a FID detector and a capillary column (19095N-123HP-INNOWAX; 30 × 0.530 mm × 1.00 μm; J&W Scientific, USA) [45]. The samples were centrifuged at 10,000 rpm and filtered through a 0.45 μm membrane filter, before analysis. Soluble carbohydrate and protein of filtrate samples were analyzed immediately.

The energy and coulombic efficiency were calculated to characterize the performance of MEC reactor. Columbic efficiency indicated the electron recovery from substrates, which was defined by the ratio of coulomb recovery to the total coulombs in the substrate. The coulomb recovery can be calculated by the equation *Q* = ∫*i*·∆*t*, where *i* is the current of the external circuit. The total coulombs can be calculated by the equation $Q_{t} = ({{COD_{in}}} - {{COD_{eff}}}) \cdot V \cdot F \cdot b/M_{{O_{2} }}$, where *F* represents the Faraday constant, 96,485 C/mol; $M_{{O_{2} }}$ is the molar mass of oxygen, 32 g/mol; *b* is the complete oxidation requirement of electron per mole oxygen and b is 4 mol-e^−^/mol; COD is measured from influence and effluence of SFL.

### DNA extraction and Illumina sequencing

Microbial community samples were collected from fermentation liquid on the 3rd day of fermentation and anode biofilm at the end of MEC operations. PowerSoil DNA Isolation Kit (Mo Bio Laboratories, Carlsbad, CA, US) was used to extract the genomic DNA of the samples, according to the manufacturer’s instructions. The quantity and quality of the extracted DNA were checked by measuring its absorbance at 260 and 280 nm, using a spectrophotometer. Amplicon was constructed for Illumina sequencing, using bacterial fused primers 341F (5′-CCTACACGACGCTCTTCCGATCTN (barcode) CCTACGG-GNGGCWGCAG-3′) and 805R (5′-GACTGGAGTTCCTTGGCACCCGAGAATTCCA (barcode) GACTACHVGGGTATCTAATCC-3′) for the V3–V4 region of the 16S rRNA gene [74]. PCRs were performed in a total volume of 50 μL, containing 1 × PCR buffer, 1 mM dNTPs, 5 μM each primer, 1 U Plantium Taq, and 10 ng of template DNA. The PCR amplification program contained an initial denaturation at 94 °C for 3 min, followed by 5 denaturing cycles at 94 °C for 30 s, annealing at 45 °C for 20 s, and extension at 65 °C for 30 s, then followed by 20 cycles of denaturing at 94 °C for 20 s, annealing at 55 °C for 20 s, and extension at 72 °C for 30 s, finally followed by a final extension at 72 °C for 5 min.

Before sequencing, PCR products of different samples were normalized in equimolar amounts in the final mixture, which was used to construct the PCR amplicon libraries. Sequencing was carried out on an Illumina HiSeq 2000, and the raw sequences have been deposited in the NCBI Short Read Archive (SRA) database, under the accession number SRR1532554. With similarity set at 97 % and a confidence threshold of 95 %, the obtained sequences were phylogenetically allocated down to the phylum, class, and genus level with the MOTHUR program (http://www.mothur.org/wiki/Main_Page). To define the relative abundance of a given phylogenetic group, the number of sequences affiliated to that group were divided by the total number of obtained sequences. The results were used for the analysis and comparison of microbial community structure differences. The Cytoscape network layout used in this work was made by Cytoscape 3.2.1, using detected OUTs as node connectivity to illustrate groups and inter-group relationships [75, 76].

## Additional file

10.1186/s13068-016-0493-2 **Table S1.** The main contents of sludge fermentative liquid on the 3rd day, after different pretreatments. **Table S2.** The main characteristics of raw waste activated sludge. **Table S3.** The detected number of sequences, OTUs and diversity. **Fig. S1.** Particle size distribution in sludge, obtained through different pretreatments. Control: sludge without treatment; A: Alkaline; F: Freeze/thaw; U: Ultrasonic treatment. **Fig. S2.** Lysis ratio of increased SCOD to TCOD in sludge, after different pretreatments. **Fig. S3.** MEC reactor setup: COD removal and Coulombic efficiency obtained by feeding artificial wastewater. **Fig. S4.** VFAs content in different sludge fermentative liquids. **Fig. S5.** Main contents of COD removal (Polysaccharide, protein, VFAs) in MECs fed with different sludge fermentative liquids. **Fig. S6.** Current change in the last three batch MECs fed with different sludge fermentative liquids. **Fig. S7. **Rarefaction curves (a) and Shannon diversity (b) base on pyrosequencing of bacterial communities. The OTUs were defined by 3 % distance. **Fig. S8.** Taxonomic classification of bacterial communities of sludge fermentative liquid at the phylum (a), class (b) and genus (c) levels. Relative abundance was defined as the number of sequences per sample. **Fig. S9.** The setup of single chamber microbial electrolysis cell (MEC). **Fig. S10.** A flow schematic representation of experimental methodology and reactor setup.

## Abbreviations

MEC: microbial electrolysis cell
AD: anaerobic digestion
WAS: waste activated sludge
BESs: bioelectrochemical systems
COD: chemical oxygen demand
SCOD: soluble chemical oxygen demand
TCOD: total chemical oxygen demand
VFAs: volatile fatty acids
LB-EPS: loosely bound extracellular polymeric substances
TB-EPS: tightly bound extracellular polymeric substances
SFL: sludge fermentation liquid
PBS: phosphate buffer solution
OTUs: operational taxonomic units
ACE: abundance-based coverage estimator
VSS: volatile suspended solids
DOMs: dissolved organic matters
