# Supplementary material for: Microbial network for waste activated sludge cascade utilization in an integrated system of microbial electrolysis and anaerobic fermentation
Source: Biotechnol Biofuels. 2016 Apr 2;9:83. doi: 10.1186/s13068-016-0493-2 (PMC4818858; doi:10.1186/s13068-016-0493-2)
Supplement: Supplementary file 1 — 10.1186/s13068-016-0493-2 Table S1. The main contents of sludge fermentative liquid on the 3rd day, after different pretreatments. Table S2. The main characteristics of raw waste activated sludge. Table S3. The detected number of sequences, OTUs and diversity. Fig. S1. Particle size distribution in sludge, obtained through different pretreatments. Control: sludge without treatment; A: Alkaline; F: Freeze/thaw; U: Ultrasonic treatment. Fig. S2. Lysis ratio of increased SCOD to TCOD in sludge, after different pretreatments. Fig. S3. MEC reactor setup: COD removal and Coulombic efficiency obtained by feeding artificial wastewater. Fig. S4. VFAs content in different sludge fermentative liquids. Fig. S5. Main contents of COD removal (Polysaccharide, protein, VFAs) in MECs fed with different sludge fermentative liquids. Fig. S6. Current change in the last three batch MECs fed with different sludge fermentative liquids. Fig. S7. Rarefaction curves (a) and Shannon diversity (b) base on pyrosequencing of bacterial communities. The OTUs were defined by 3 % distance. Fig. S8. Taxonomic classification of bacterial communities of sludge fermentative liquid at the phylum (a), class (b) and genus (c) levels. Relative abundance was defined as the number of sequences per sample. Fig. S9. The setup of single chamber microbial electrolysis cell (MEC). Fig. S10. A flow schematic representation of experimental methodology and reactor setup. [file 13068_2016_493_MOESM1_ESM.pdf]

Supporting information for

Microbial network for waste activated sludge cascade utilization in an integrated system of microbial electrolysis and anaerobic fermentation

Wenzong Liu[1], Zhangwei He[2], Chunxue Yang[2], Aijuan Zhou[3], Zechong Guo[2], Bin Liang[1], Cristiano Varrone[4], Ai-Jie Wang [1,2*]

Table S1 The main contents of sludge fermentative liquid on the 3rd day, after different pretreatments

Item / Treatment	SCOD (mg/L)	Polysaccharide (mg/L)	Protein (mg/L)	VFAs (mg COD/L)	Conductivity (mS/cm)	pH
Control	452	24.97	72.67	332.75	1.41	6.86
Alkaline	7690	559.19	1749.33	5304.06	6.23	7.93
Freeze/thaw	1760	47.79	372.67	1286.80	1.96	6.76
Ultrasonic	3461	209.93	572.67	2601.39	2.63	6.47

The contents includes SCOD, Polysaccharide, Protein, VFAs, Conductivity and pH in comparison among pretreated WAS fermentative liquid

Table S2 The main characteristics of raw waste activated sludge

Parameter	Value [a]
pH	6.86±0.21
TSS (total suspended solids, g/L)	25.4 ±0.6
VSS (volatile suspended solids, g/L)	13.6 ± 0.7
SCOD (soluble chemical oxygen demand, mg/L)	147 ± 22
TCOD (total chemical oxygen demand, mg/L)	19933 ± 198
VFAs (as COD, mg COD/L)	102 ± 14
Soluble carbohydrate (as COD, mg COD/L)	11 ± 2
Soluble protein (as COD, mg COD/L)	20 ± 3
Conductivity (mS/cm)	1.21 ± 0.12

a average value and standard deviation.

The basic property of WAS used in the experiment, which was taken from local waste water treatment plant, including pH, TSS (total suspended solids, g/L), VSS (volatile suspended solids, g/L), SCOD (soluble chemical oxygen demand, mg/L), TCOD (total chemical oxygen demand, mg/L), VFAs (as COD, mg COD/L), Soluble carbohydrate (as COD, mg COD/L), Soluble protein (as COD, mg COD/L), and Conductivity (mS/cm).

Table S3 The detected number of sequences, OTUs and diversity

Sample ID	Seq No.	OTUs	Shannon index	ACE index	Chao1 index
Raw	24212	5002	6.96	22447	13299
F-control	24405	4356	6.95	14506	10047
F-a	23490	3865	6.64	11878	8414
F-f	25659	4123	6.69	12256	8693
F-u	25393	2393	5.25	6831	4944
Mec	22217	2341	5.39	6633	4694
M-control	20231	4079	6.61	13063	9153
M-a	24936	3889	6.21	12192	8018
M-f	27821	4517	6.58	15040	10098
M-u	26397	3559	6.02	12061	7940

F: fermentation communities. M: MEC biofilm communities.
Sludge pretreatment: a) alkaline; f) freeze-thaw; u) ultrasonic; control) without pretreatment.

The overall detected sequences in Raw WAS, suspended solution and biofilm in all reactors. The diversity of all samples were calculated as Shannon index, ACE index and Chao1 index.

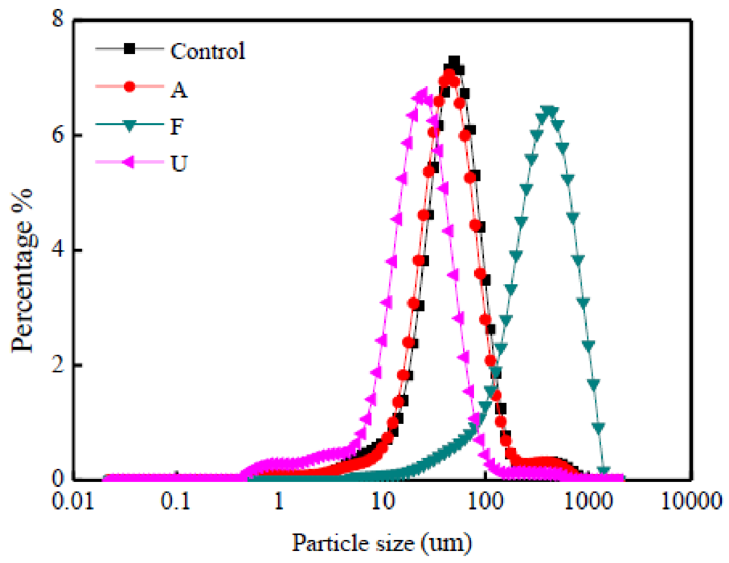

Fig. S1 Particle size distribution in sludge, obtained through different pretreatments. Control: sludge without treatment; A: Alkaline; F: Freeze/thaw; U: Ultrasonic treatment.

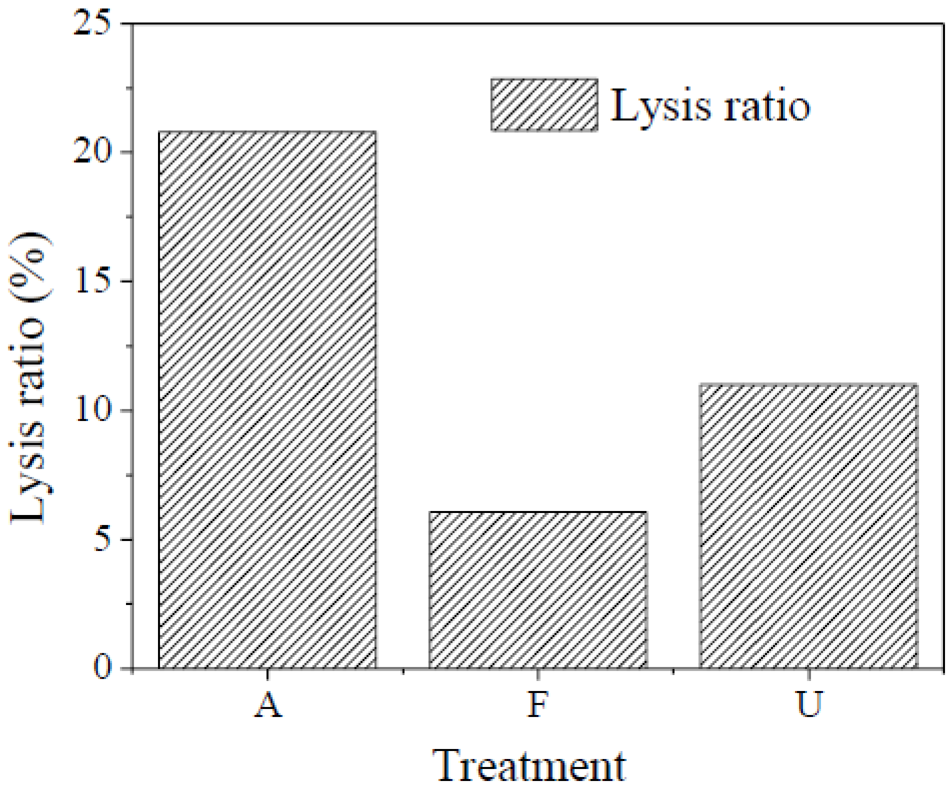

Fig. S2 Lysis ratio of increased SCOD to TCOD in sludge, after different pretreatments

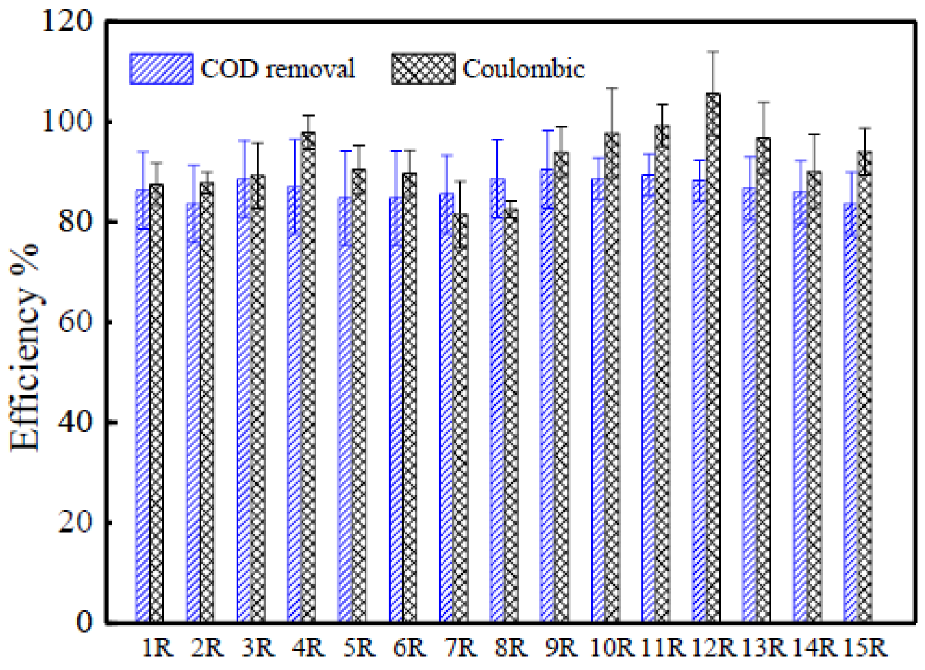

Fig. S3 MEC reactor setup: COD removal and Coulombic efficiency obtained by feeding artificial wastewater

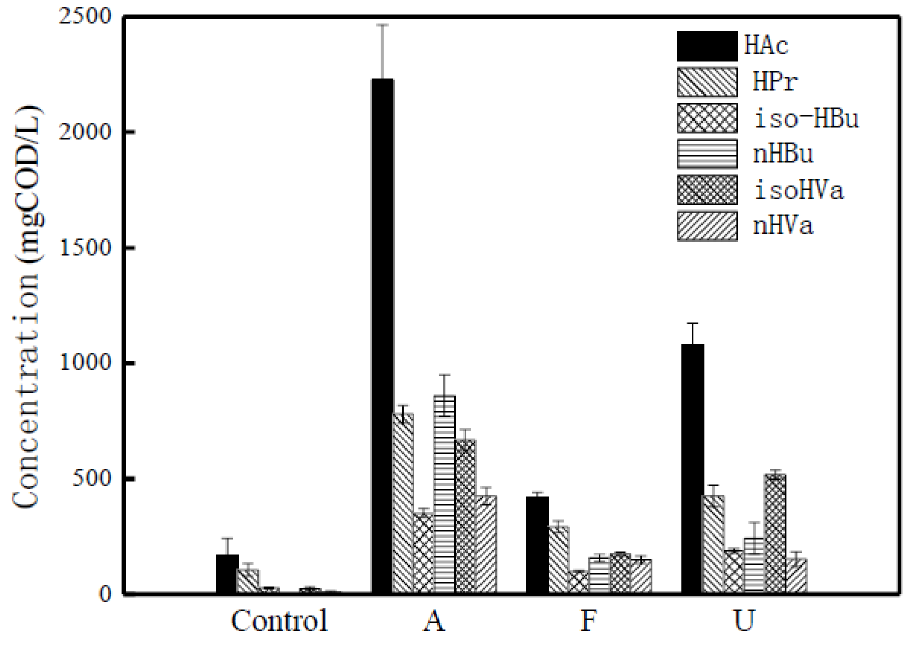

Fig. S4 VFAs content in different sludge fermentative liquids

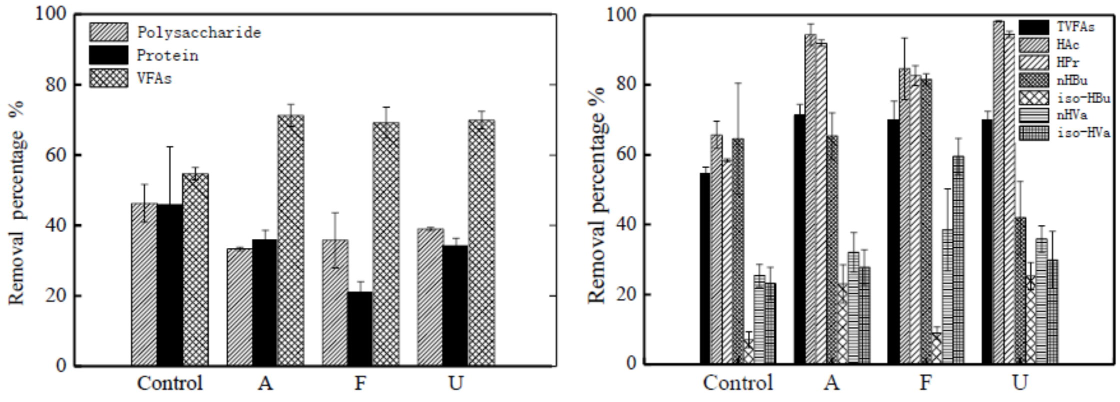

Fig. S5 Main contents of COD removal (Polysaccharide, protein, VFAs) in MECs fed with different sludge fermentative liquids

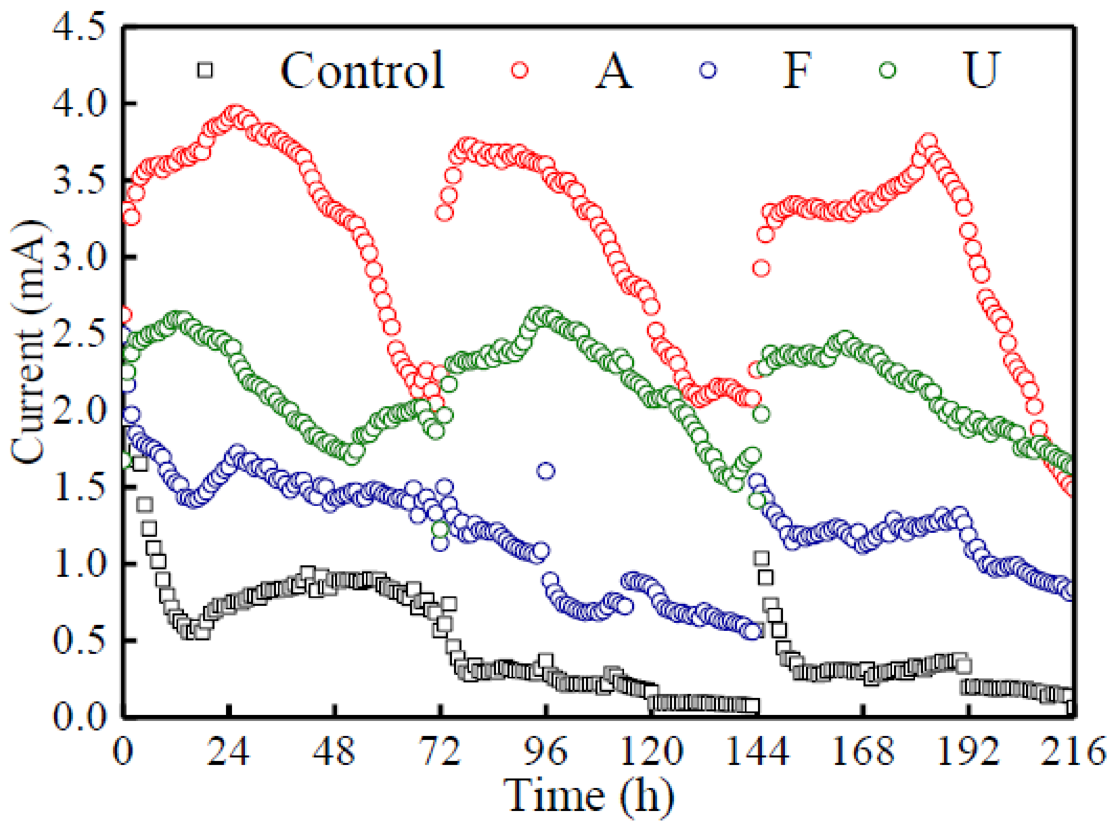

Fig. S6 Current change in the last three batch MECs fed with different sludge fermentative liquids.

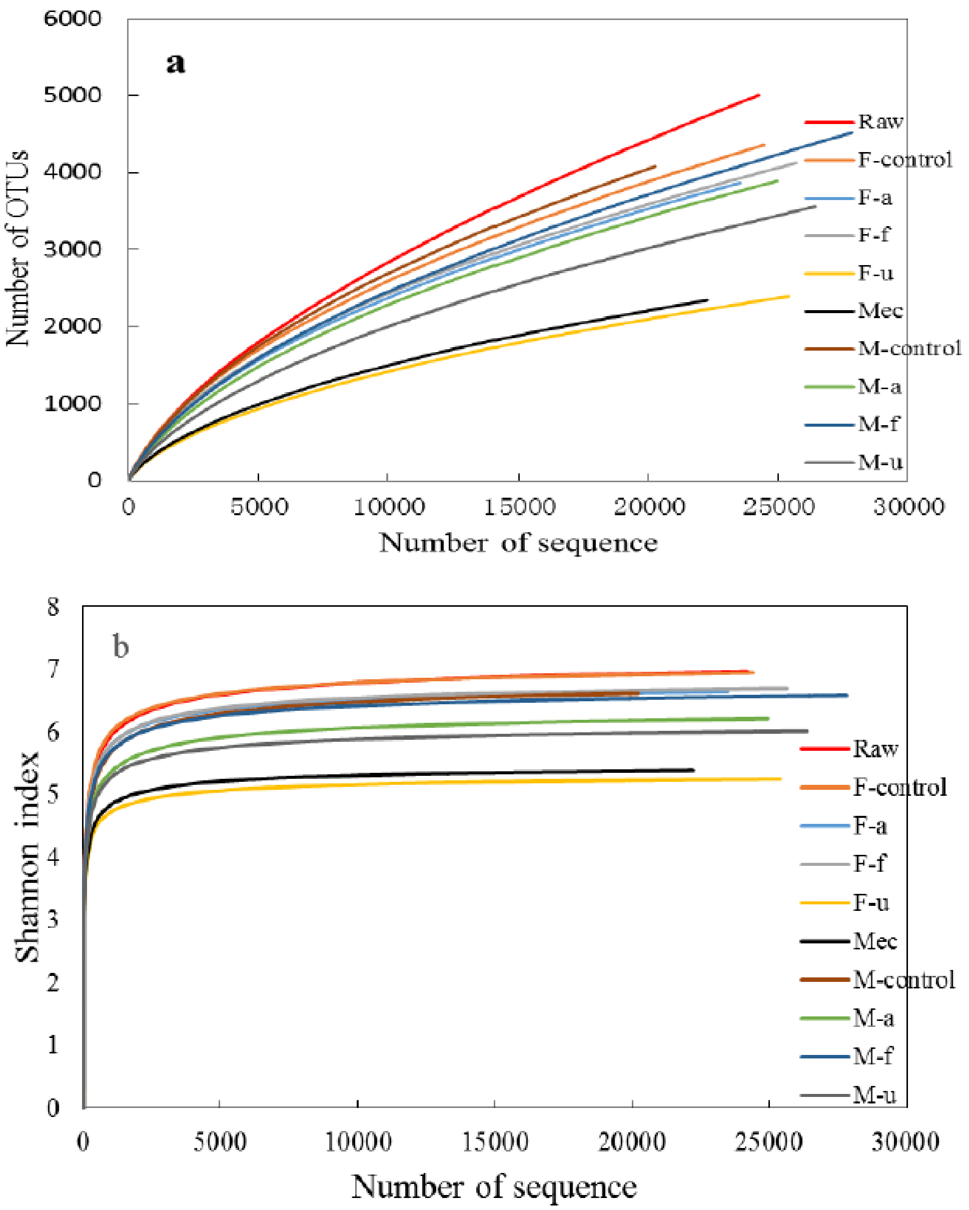

Fig. S7 Rarefaction curves (a) and Shannon diversity (b) base on pyrosequencing of bacterial communities. The OTUs were defined by 3% distance.

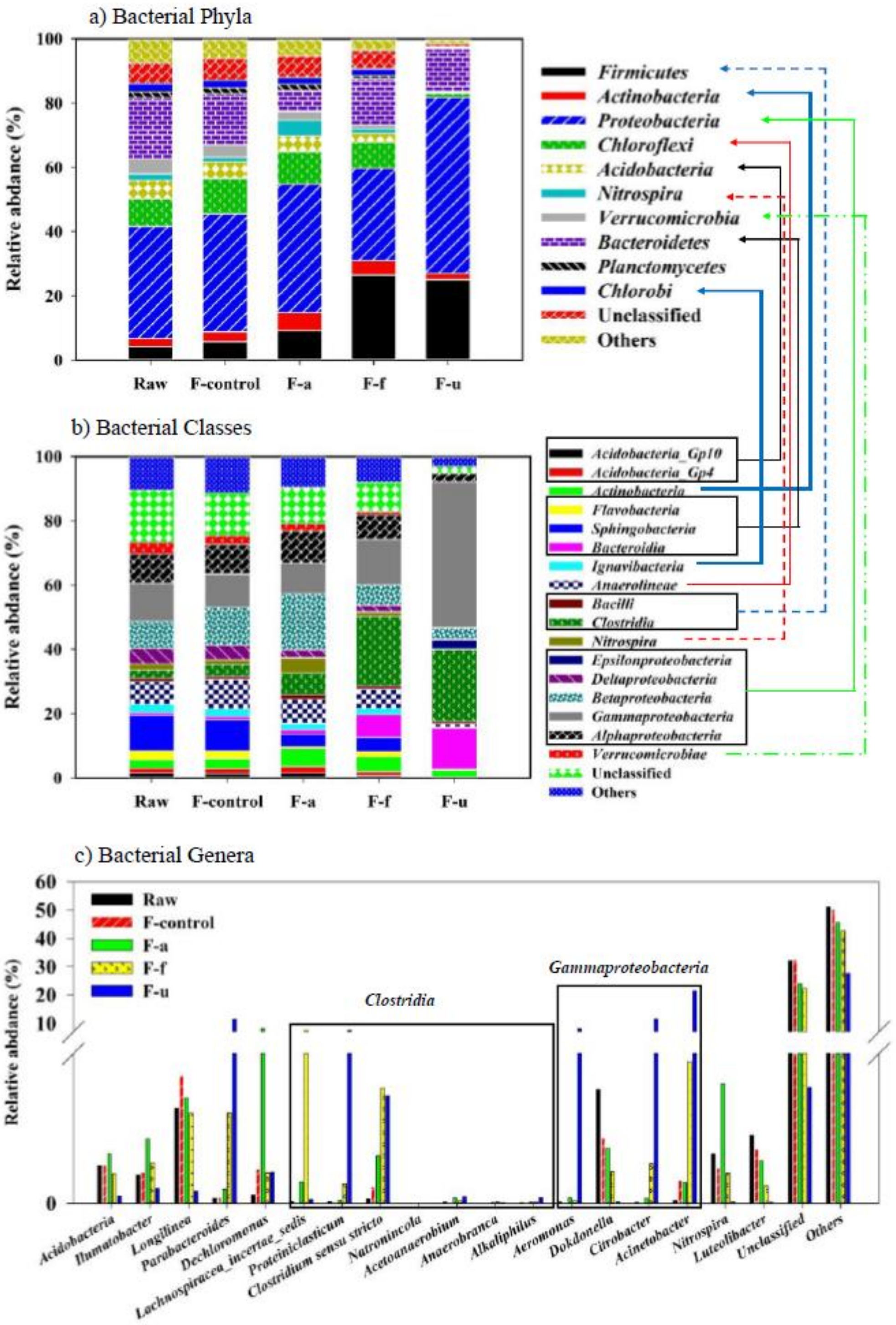

Fig. S8 Taxonomic classification of bacterial communities of sludge fermentative liquid at the phylum (a), class (b) and genus (c) levels. Relative abundance was defined as the number of sequences per sample.

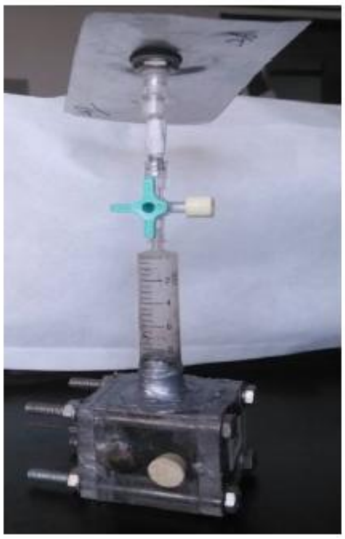

Fig. S9 The setup of single chamber microbial electrolysis cell (MEC).

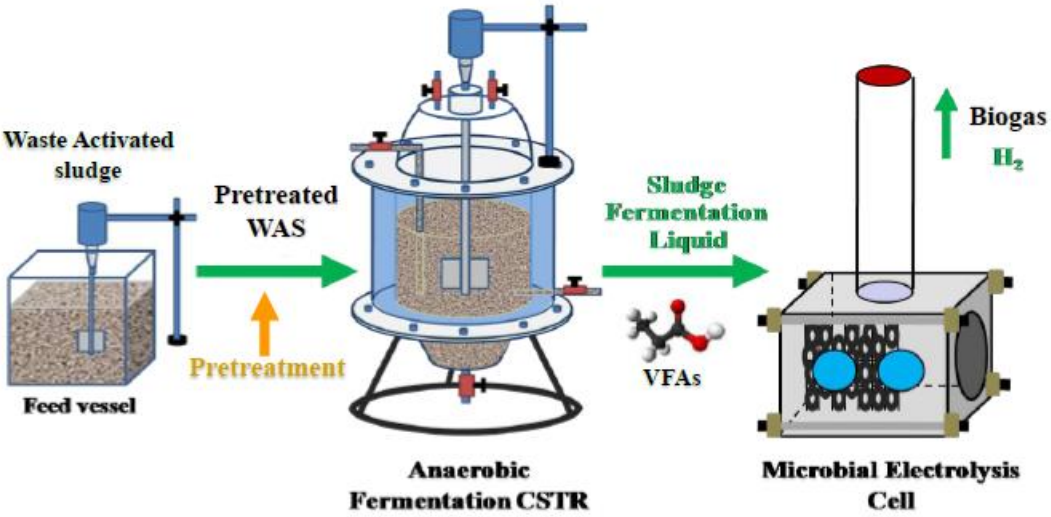

Fig. S10 A flow schematic representation of experimental methodology and reactor setup
